# Herpes zoster encephalitis presenting as multiple cerebral hemorrhages – a rare presentation: a case report

**DOI:** 10.1186/1752-1947-7-155

**Published:** 2013-06-18

**Authors:** Amrish Saxena, Benjamine Khiangte, Iadarilang Tiewsoh, Ulhas N Jajoo

**Affiliations:** 1 Department of Medicine, Mahatma Gandhi Institute of Medical Sciences, Sevagram, Dist. Wardha, Maharashtra, India

**Keywords:** Diagnosis, Herpes zoster virus, Intracerebral hemorrhage, Vasculopathy, Viral encephalitis

## Abstract

**Introduction:**

An infection by herpes zoster virus is a common and important cause of encephalitis. Herpes zoster virus encephalitis if not treated promptly can result in significant morbidity and mortality. The diagnosis of herpes zoster virus encephalitis is based on clinical history, examination, neuroradiological imaging (magnetic resonance imaging and/or computed tomography scan), cerebrospinal fluid analysis and identification of the pathogen in cerebrospinal fluid by polymerase chain reaction amplification and/or anti-herpes zoster virus immunoglobulin G antibody in cerebrospinal fluid. Although ischemic intracerebral infarcts in patients with herpes zoster virus encephalitis or vasculopathy are reported in the literature, multiple intracerebral hemorrhages as a complication of herpes zoster virus encephalitis in an immunocompetent individual are extremely rare.

**Case presentation:**

A 40-year-old Indian man presented with an acute history of four episodes of seizures, fever, headache, drowsiness, focal neurological deficits and vesicular eruptions over the abdomen in a typical dermatomal distribution. His head computed tomography scan revealed multiple cerebral hemorrhages. Investigations (positive ratio between the cerebrospinal fluid/serum quotients for anti-herpes zoster virus immunoglobulin G and total immunoglobulin G antibodies) established its infective origin due to herpes zoster virus. He developed bilateral pneumonia during the hospital course. He had an excellent recovery following a 2 weeks’ course of intravenous acyclovir.

**Conclusion:**

Herpes zoster virus encephalitis or vasculopathy is a rare cause of multiple intracerebral hemorrhages and must be considered in the differential diagnosis of patients presenting with an acute history of fever, altered consciousness, and focal neurologic deficits with history of a typical herpetic rash. Its prompt recognition and treatment could alter the course of illness.

## Introduction

Herpes zoster virus (HZV) infection is associated with neurological complications such as encephalitis, aseptic meningitis, meningoencephalitis, acute cerebellar ataxia, leukoencephalopathy, cranial nerve palsies, Ramsay Hunt syndrome, postherpetic neuralgia, radiculitis and myelitis. The frequency of HZV as a cause of encephalitis is variable, ranging from as low as 5% to as high as 15% in different series [1,2]. Cases of intracerebral hemorrhagic lesion in patients with herpes simplex virus (HSV) encephalitis are described in the literature [3,4]. Herpes zoster vasculopathy presenting as intracerebral hemorrhage is a very rare entity [5]. Although multifocal ischemic intracerebral infarcts in patients with HZV encephalitis or vasculopathy are reported in the literature, multiple intracerebral hemorrhages as a complication of HZV encephalitis in an immunocompetent individual are extremely rare [6,7]. We report here an immunocompetent patient with multiple intracerebral hemorrhages as a complication of HZV encephalitis, who also had concurrent herpes zoster rash in a dermatomal distribution over his trunk and bilateral pneumonia.

## Case presentation

**Figure 1:**
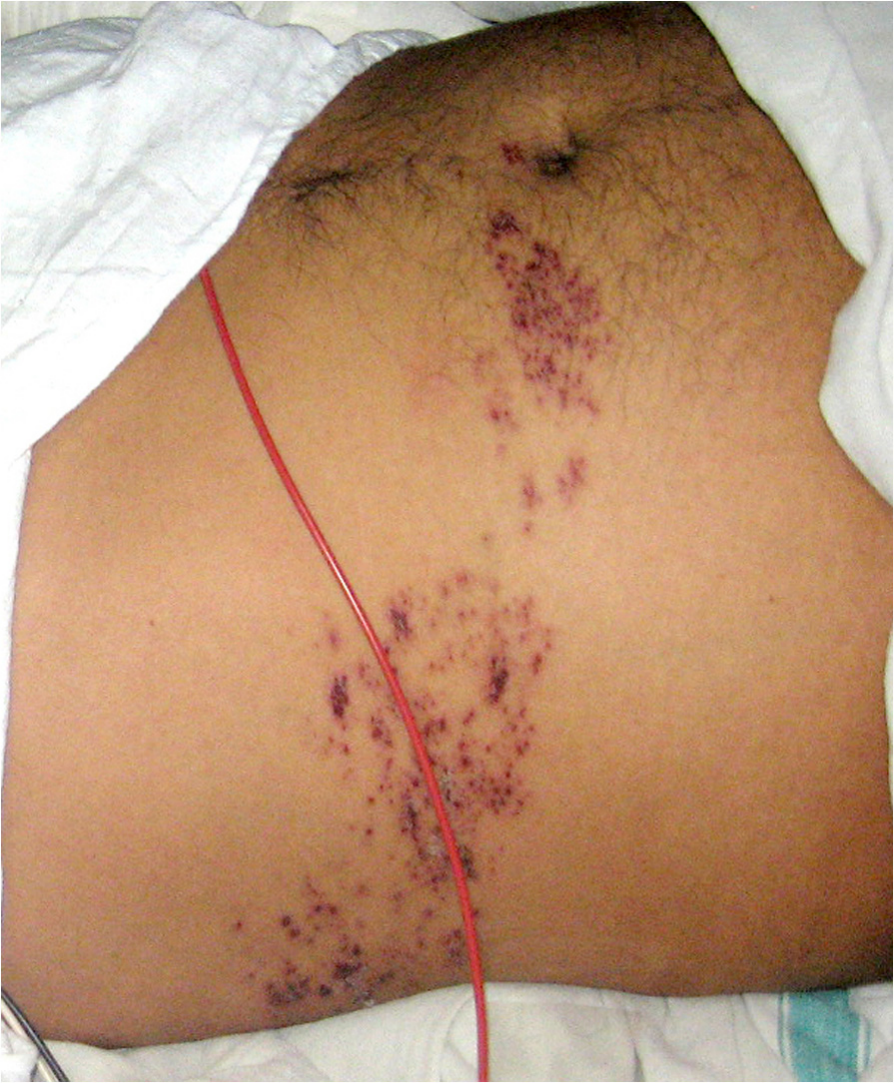
Herpes zoster rash over abdomen in a dermatomal distribution.

A 40-year-old Indian man presented with four episodes of generalized tonic–clonic seizures with a history of fever followed by headache and drowsiness since 1 day. There was no history of head injury preceding the onset of this illness. The history of the patient was gathered from his family members. On general physical examination, he was febrile (39.4°C), anicteric and drowsy. He had a pulse of 112 beats per minute, blood pressure of 120/70mmHg, and a respiratory rate of 16 breaths per minute. Vesicular eruptions on an erythematous base were present over the right side of his abdomen and back in a dermatomal distribution (T10; Figure 1). No lymphadenopathy was present. At presentation, he was stuporous, irritable and not responding to oral commands. His Glasgow Coma Score (GCS) was 9: eye opening, verbal response and motor response were 2, 2 and 5, respectively (E2V2M5).

His neurological examination revealed paucity of movement on the left side, and brisk deep tendon reflexes with left plantar extensor response. The pupils were equal and normally reactive to light. No signs of meningeal irritation were present. Fundus examination and other system examination revealed no abnormality. He had no history of seizures prior to the present incident. A history of hypertension and diabetes mellitus were not present.

Examination of peripheral blood smear failed to demonstrate any malarial parasite. The result of a rapid malaria antigen test (histidine-rich protein-II and plasmodium lactate dehydrogenase) was negative. The cerebrospinal fluid (CSF) examination revealed: lymphocytes (80/mm^3^) with presence of red blood cells (20/mm^3^), protein 90mg/dL, and glucose 108mg/dL. There was no organism in Gram and Ziehl–Neelsen staining of the CSF. His hemogram showed a total leukocyte count of 12,700/μL (with differential of 81% polymorphonuclear leukocytes, 12% lymphocytes, 7% monocytes). His platelet count, bleeding and coagulation profile were within normal limits. The results of the human immunodeficiency virus (HIV) tests were negative by both enzyme-linked immunosorbent assay (ELISA) and rapid HIV test for HIV-1 and HIV-2 antibodies.

He was empirically treated with intravenous acyclovir 500mg every 8 hours and antibiotics (intravenous ceftriaxone 2g every 12 hours) for the possible diagnosis of acute infective encephalitis or meningoencephalitis most likely viral in origin. He was given a loading dose of phenytoin sodium intravenously and then placed on 100mg intravenously every 8 hours to control seizures.

**Figure 2:**
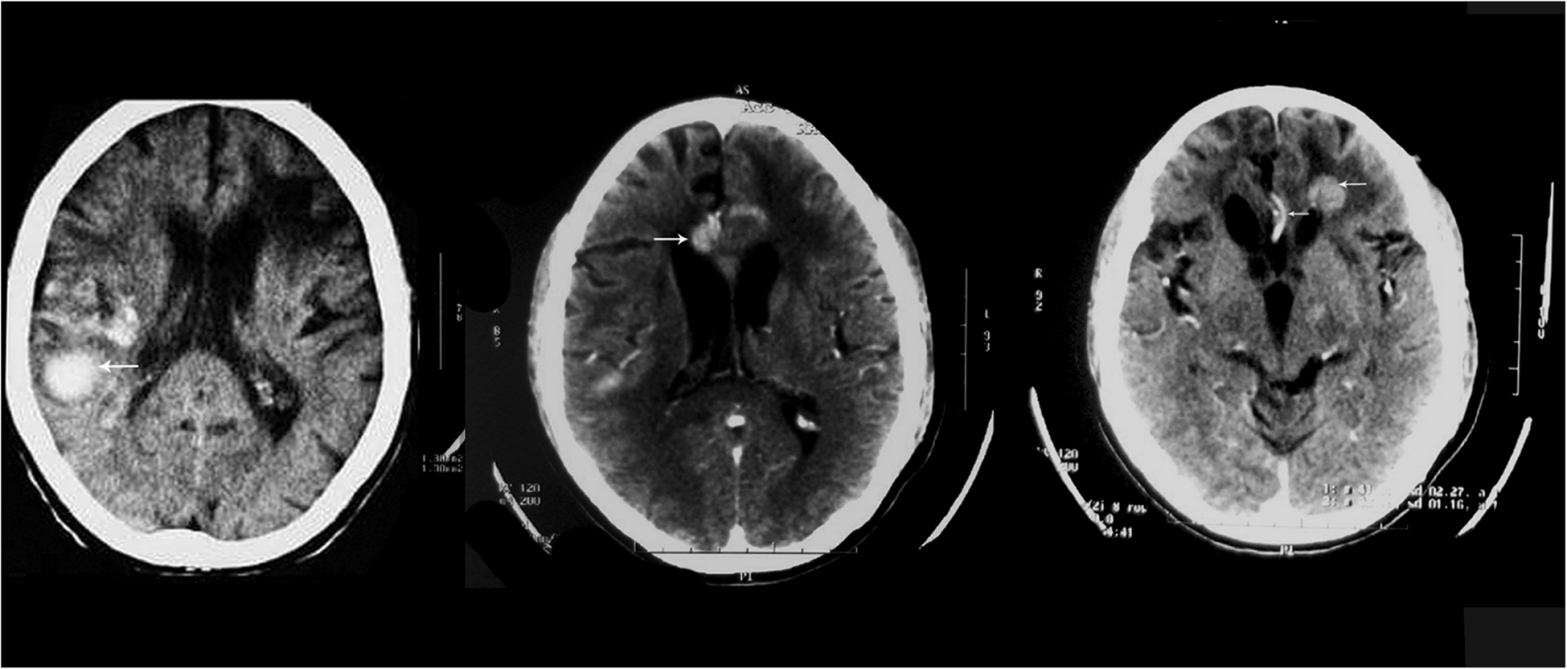
Head computed tomography scan showing multiple intraparenchymal hemorrhages (hyperdense areas) with surrounding hypodensities in the left frontal, right parietal and corpus callosum regions (arrows).

His head computed tomography (CT) scan with contrast showed multiple intraparenchymal hemorrhages (hyperdense lesions) with surrounding hypodensities in the left frontal, right parietal and corpus callosum regions (Figure 2). No abnormal contrast enhancement was seen. Magnetic resonance imaging (MRI) of his brain also revealed features of intraparenchymal hemorrhages with no abnormal contrast enhancement in the above-mentioned sites. MRI angiography did not reveal any aneurysm or vascular malformation or segmental narrowing. CSF polymerase chain reaction was negative for HSV-1 and HSV-2. Anti-toxoplasma immunoglobulin (Ig) G, IgM antibody and antinuclear antibody were found to be negative by ELISA.

**Figure 3:**
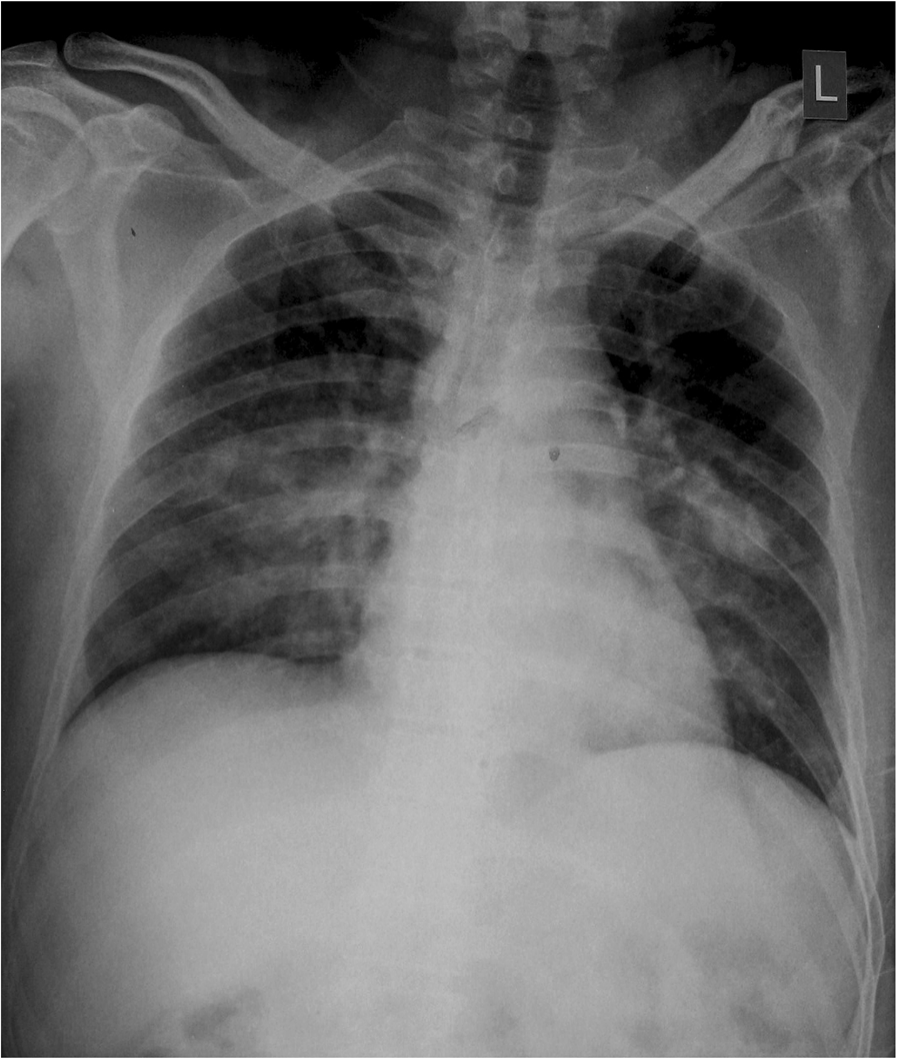
Chest radiograph showing bilateral non-homogenous fluffy infiltrates.

On the second day of hospitalization, he developed acute onset breathlessness. His chest examination revealed crackles bilaterally in the infrascapular region. He was mechanically ventilated for 3 days for type-I (hypoxemic) respiratory failure. A chest radiograph showed bilateral non-homogenous fluffy infiltrates (Figure 3). The differential diagnosis kept for his lung lesion was viral pneumonia (varicella pneumonia) in view of his skin lesions and encephalitis or acute respiratory distress syndrome (ARDS). Intravenous dexamethasone 8mg every 8 hours and levofloxacin 750mg every 24 hours were added on day 2 of hospitalization in view of ARDS.

On day 5, mechanical ventilatory support was withdrawn and his GCS improved to 13 (E4V3M6). He was confused and dysarthric. His motor power in the left upper and lower limbs was 2 out of 5. He was found to have memory disturbances, behavioral changes, disorganized speech and apraxia during the hospital course. His neurologic function, motor power, mental status and speech partially recovered gradually during the hospital stay. Dexamethasone was given for a period of 7 days and acyclovir for 14 days (started on day 2 of hospitalization).

**Figure 4:**
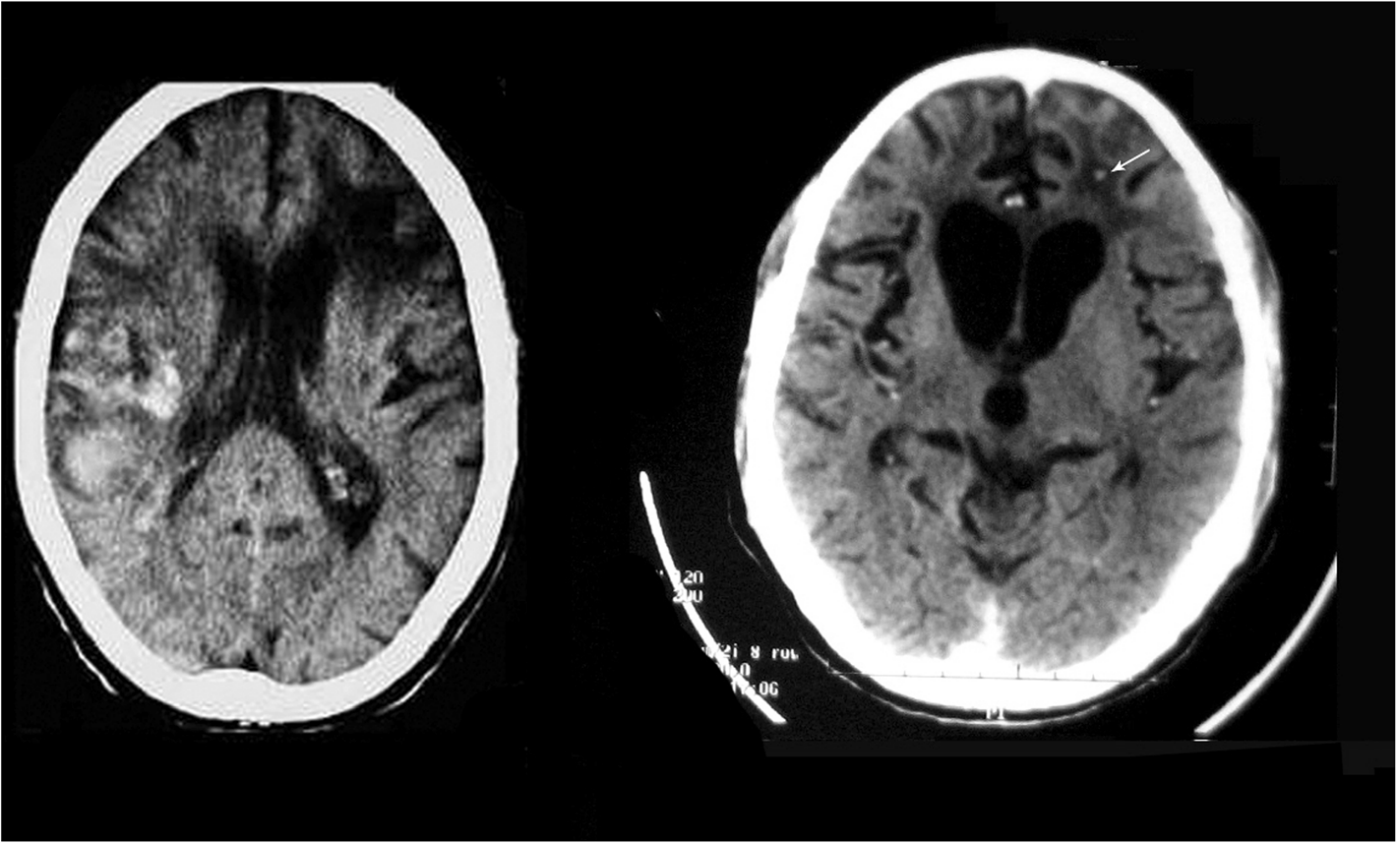
**Repeat head computed tomography on the 15**^**th **^**day of admission revealing resolving intraparenchymal hemorrhages (arrow).**

Repeat head CT scan after 15 days revealed partial resolution of intraparenchymal hemorrhages in the frontal, right parietal and corpus callosum regions (Figure 4). On the 15^th^ day of hospitalization, his anti-HZV antibody index (ratio between the CSF/serum quotients for anti-HZV IgG and total IgG antibodies) was found to be positive in repeated lumbar puncture, which is indicative of local HZV specific antibody synthesis in the central nervous system (CNS). He was diagnosed as a case of HZV encephalitis with multiple intraparenchymal hemorrhages and varicella pneumonia. The patient was discharged with partial neurological recovery after 2 weeks of treatment with acyclovir. The patient was able to feed himself, to walk with assistance and to speak simple words. His motor strength in the left upper and lower limbs improved to 4 out of 5.

## Discussion

The viruses commonly responsible for causing encephalitis in immunocompetent patients are human HSV-1, HZV or varicella zoster virus, Epstein–Barr virus, mumps, measles, and enteroviruses [1]. The neurological complications caused by HZV occur due to vasculitis affecting small or large vessels [8,9]. HZV vasculopathy affects both immunocompetent and immunocompromised patients, and can be either unifocal or multifocal. HZV vasculopathy may involve large vessels in immunocompetent patients, ranging from necrotizing granulomatous arteritis to chronic vasculitis and thrombosis of cerebral vessels. HZV vasculopathy may involve small vessels and ependyma in immunocompromised patients, especially those with acquired immune deficiency syndrome or malignancy or the elderly [10,11]. In our case, the patient was not immunocompromised and had no evidence of underlying malignancy. In a past report, both HZV deoxyribonucleic acid (DNA) and HZV-specific antigen were demonstrated in the media of cerebral arteries of a patient who died after HZV vasculopathy [12]. Cerebral angiography in a patient of HZV encephalitis reported by Jain *et al*. revealed multiple short segments of narrowing and beading involving the small- and medium-sized vessels, suggestive of vasculitis [5]. MRI angiography in our patient did not reveal any segmental narrowing or beading.

In a previous case series reported by Nagel *et al*., the detection of anti-HZV IgG antibody in CSF was a more sensitive indicator of HZV vasculopathy than detection of HZV DNA, which becomes detectable in CSF generally after 2 weeks [13,14]. HZV IgG antibody becomes detectable in CSF generally after 2 weeks, which lasts for months. Hence, in our case a lumbar puncture was repeated on the 15^th^ day of hospitalization for the detection of intrathecal anti-HZV IgG antibody. Similarly in an another study, it was found that testing for anti-HZV IgG antibody identified more cases of HZV vasculopathy [14].

An absence of history of head trauma, normal coagulation and hematological profile, and hemorrhages at unusual sites exclude the other possible causes like traumatic hemorrhage, coagulation or hematological disorders and hypertensive hemorrhage respectively. The resolution of the radiological lesions in the head CT scan repeated after 15 days ruled out the possibility of a brain tumor or metastases. No abnormal contrast enhancement of the lesions in the CT and MRI scans of his brain also make the diagnosis of brain tumor or metastases or arteriovenous malformations unlikely. In our patient, clinical presentation (fever, headache, seizures, focal neurologic deficits and typical vesicular eruptions), CSF findings, and the presence of anti-HZV IgG antibodies established the diagnosis of HZV encephalitis.

## Conclusion

HZV encephalitis or vasculopathy is a rare cause of multiple intracerebral hemorrhages and must be considered in the differential diagnosis of patients presenting with an acute history of fever, altered consciousness, and focal neurologic deficits with history of a typical herpetic rash. Its prompt recognition and treatment could alter the course of illness.

## Consent

Written informed consent was obtained from the patient for publication of this case report and any accompanying images. A copy of the written consent is available for review by the Editor-in-chief of this journal.

## Competing interests

The authors declare that they have no competing interests.

## Authors’ contributions

AS performed patient care, and conducted the literature review, data analysis, and drafting of the manuscript. BK, IT, and UNJ performed patient care, and revised the manuscript. All authors read and approved the final manuscript.
